# Phylogenetic relationship and characterization of the complete mitochondrial genome sequence of *Smerinthus planus* (Lepidoptera: Sphingidae)

**DOI:** 10.1080/23802359.2022.2080014

**Published:** 2022-06-02

**Authors:** Yin-Feng Meng, Chao-Fan Chen, Yi-Xin Huang, Xu Wang, Bo Zhang

**Affiliations:** a College of Biology Pharmacy and Food Engineering, Shangluo University, Shangluo, China; b Shanxi Forestry and Grassland Bureau, Taiyuan, China; c School of Ecology and Environment, Collaborative Innovation Center of Recovery and Reconstruction of Degraded Ecosystem in Wanjiang Basin Co-founded by Anhui Province and Ministry of Education, Anhui Normal University, Wuhu, China; d College of Life Sciences, Anhui Provincial Key Laboratory of the Conservation and Exploitation of Biological Resources, Anhui Normal University, Wuhu, China

**Keywords:** Bombycoidea, mitochondrial genome, phylogenetic relationship, Dabie Mountain

## Abstract

In this study, the complete mitochondrial genome of *Smerinthus planus* Walker, 1856 was sequenced and analyzed. This mitochondrial genome is circular, 15,375 bp long, and includes 37 typical metazoan mitochondrial genes (13 protein-coding genes (PCGs), two ribosomal RNA genes, and 22 transfer RNA genes) and an A + T-rich region. Nucleotide composition is highly biased toward A + T nucleotides (80.1%). All 13 PCGs initiate with the standard start codon of ATN and terminate with the typical stop codon TAA/TAG. Phylogenetic analyses were performed using amino acids of 13 PCGs which shows that *S. planus* is closely related to *Barbourion lemaii*.

The hawk moths (Sphingidae: Lepidoptera) belong to the lepidopteran group Macrolepidoptera, which are relatively large-sized moths, including 1450 species (van Nieukerken et al. 2011). Mitogenomes were widely used in reconstructing taxa in Lepidoptera, and exhibited advantage (Wang et al. 2019; Chen et al. 2020). Currently, complete mitogenome sequences of Sphingidae are very limited. Thus, more mitogenome sequences of Sphingidae would be helpful to conduct mitogenome-based phylogeny and to understand the genomic characteristics of the family. The hawk moth, *Smerinthus planus* Walker, 1856 is distributed in western and northern Asia. The mitogenome sequence of *S. planus* so far remains unknown. Therefore, we sequenced the complete mitochondrial DNA genome of *S. planus* to provide more comprehensive data for this species and also for its relationship within the family Sphingidae.

*S. planus* was collected from the Dabie Mountain, Lu'an City, Anhui Province, China (31°13′08″N, 116°20′19″E) in May 2021 and deposited in the Entomological Museum, College of Life Sciences, Anhui Normal University (https://biology.ahnu.edu.cn/info/1066/4123.htm, YX, Huang, huangyx@ahnu.edu.cn) under the accession no. DB20210523. All animal-related experiments were performed according to the protocols approved by the Institutional Animal Care and Use Committee of Anhui Normal University (Grant number AHNU-ET2021032). It was first identified by morphology and its male genitalia. Then, the complete mitochondrial genome of *S. planus* was determined by the Illumina platform (Andrews 2020). The raw paired reads were quality-trimmed and assembled into the complete circular mitogenome in Novoplasty 2.7.2.

The *S. planus* mitochondrial genome is 15,375 bp (GenBank accession number MZ593604) in length with a total A + T content of 80.1% that is heavily biased toward the A and T nucleotides. The overall base composition of the mitogenome was estimated to be A: 40.9%, T: 39.2%, C: 12.1%, and G: 7.8%. The *S. planus* mitochondrial genome encodes the complete set of 37 genes, which are usually found in animal mitogenomes: including 13 protein-coding genes (PCGs), 22 transfer RNA (tRNA) genes, two ribosomal RNA (rRNA) genes, and one non-coding region (the A + T-rich region).

There are 23 genes (nine PCGs and 14 tRNAs) encoded on the heavy (H) stand, and the remaining 14 (four PCGs, eight tRNAs, and two rRNAs) genes on the light (L) strand. Among the 13 PCGs in the *S. planus* mitogenome, nine PCGs (*nad2*, *nad3*, *nad6*, *cox1*, *cox2*, *cox3*, *atp6*, *atp8*, *cytb*) are encoded by the H strand, while the other four PCGs are encoded on the L strand. All 13 PCGs start with ATN and stop with traditional TAA or TAG codons, which is similar to most other insect mitogenomes (Crozier and Crozier 1993; Korkmaz et al. 2015). All 22 tRNA genes usually found in the mitogenomes of insects are present in *S. planus*. The nucleotide length of tRNA genes ranges from 64 bp (trnC) to 71 bp (trnK), and A + T content ranges from 70.4% (trnK) to 92.6% (trnE). These two rRNA genes have been identified on the L strand in the *S. planus* mitogenome.

To investigate the phylogenetic implications of the *S. planus* mitogenome in Sphingidae phylogeny, a total of 26 taxa, namely 24 Sphingidae species and two outgroups, were sampled for phylogenetic analyses. The nucleotide sequences of each PCG were aligned by MAFFT (Katoh et al. 2005), employed under the G-INS-I algorithm. This was followed by minor manual editing, mostly to limit gap introduction in partial sequences. Then, the aligned sequences were concatenated into a dataset. We analyzed the nucleotide sequences of PCGs using the maximum-likelihood (ML) on the W-IQ-Tree web server method to reconstruct the phylogenetic relationship of *S. planu*s with other Sphingidae under the best substitution models for each partition selected by W-IQ-Tree web server (Trifinopoulos et al. 2016). An ultrafast bootstrap (UFB) of 1000 replications was used in this analysis to assess branch supports.

The result showed that all the subfamilies formed monophyletic groups respectively (Figure 1), which is in accordance with the previous study (Wang et al. 2021). Sphinginae was recovered as sister to the clade formed by Smerinthinae. In Smerinthinae, *S. planus* was closely related to *Barbourion lemaii*.

**Figure 1.**
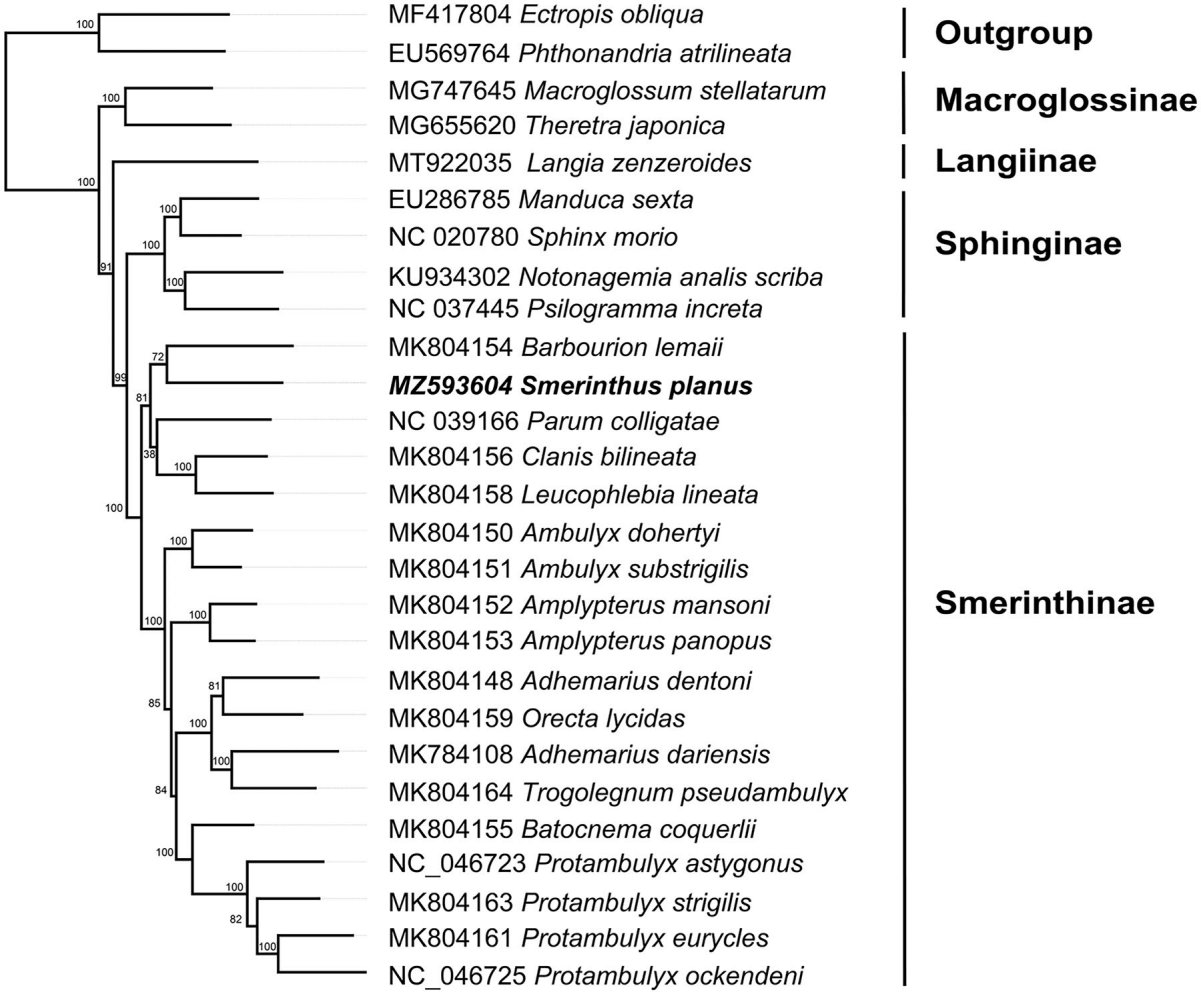
Phylogenetic relationships within Sphingidae based on the sequences of 13 protein-coding genes were performed using ML methods. The values of ultrafast bootstrap (UFB) of 1000 replications are on the nodes.

## Author contributions

Yin-Feng Meng: the conception and design, analysis and interpretation of the data, the drafting of the paper, and revising it critically for intellectual content. Chao-Fan Chen: the conception and design, analysis and interpretation of the data. Yi-Xin Huang: the conception and design, analysis and interpretation of the data. Xu Wang: the conception and design, analysis and interpretation of the data. Bo Zhang: the conception and design, analysis and interpretation of the data, the drafting of the paper, revising it critically for intellectual content and the final approval of the version to be published. All authors agree to be accountable for all aspects of the work.

## Data Availability

The data that support the findings of this study are openly available in GenBank at https://www.ncbi.nlm.nih.gov/genbank/, reference number MZ593604. The associated BioProject, Bio-Sample numbers, and SRA are PRJNA752251, SAMN20587853, and SRR15357862, respectively.
